# Editorial for “Diagnosis, Classification, and Monitoring of Pulmonary Diseases”

**DOI:** 10.3390/diagnostics15192520

**Published:** 2025-10-05

**Authors:** Paola Confalonieri, Francesco Salton, Barbara Ruaro

**Affiliations:** Pulmonology Unit, Department of Medical Surgical and Health Sciences, Hospital of Cattinara, University of Trieste, 34149 Trieste, Italy

This Special Issue offers a comprehensive overview of recent advances and innovative approaches in the field of lung disease research, emphasizing the importance of technological and conceptual innovations that are shaping our understanding of respiratory conditions. The collected studies highlight the development of novel diagnostic tools, biomarkers, and risk stratification strategies that aim to improve early detection, personalized treatment, and patient management.

Several articles focus on enhancing prognostic accuracy through the identification of biomarkers—whether in pulmonary hypertension, COPD, or lung cancer—that can predict disease progression and outcomes more effectively [1,2,3,4]. Others explore cutting-edge imaging techniques, such as advanced microscopy, ultrasound, and oscillometry, which provide deeper insights into lung pathology while offering less invasive options for diagnosis and monitoring [4,5,6,7,8].

In addition, the research demonstrates progress in procedural safety and efficacy, exemplified by studies on minimally invasive pleuroscopy and real-world treatment outcomes. Emerging diagnostic modalities, like the thermal imaging of exhaled CO_2_ and microvascular assessments via non-invasive imaging, open new avenues for non-contact, rapid detection of respiratory abnormalities [4,5,6,7,8].

Importantly, the collection underscores a trend toward integrating molecular, imaging, and clinical data to foster personalized medicine approaches, ultimately aiming to improve patient outcomes across a spectrum of lung diseases [1,2,3,4]. This body of work not only advances scientific understanding but also highlights the critical role of research and innovation in addressing the complex challenges faced in respiratory healthcare.

The importance of ongoing research and studies cannot be overstated, as they serve as the foundation for the development of new diagnostic approaches that could revolutionize how we detect and manage pulmonary diseases. By leveraging innovative technologies and methodologies, researchers are paving the way for more accurate, early diagnoses that are crucial for effective intervention [4,5,6,7,8]. This progress is vital, considering the often insidious progression of many lung conditions, which can lead to delayed treatment and poorer outcomes.

Furthermore, continuous research efforts are essential for translating scientific discoveries into clinical practice, ensuring that patients benefit from the latest advancements. The integration of novel diagnostic strategies not only enhances our ability to identify diseases at earlier stages but also enables more personalized and targeted therapies, reducing side effects and increasing treatment efficacy.

Ultimately, sustained investment in research fuels innovation, leading to the discovery of new biomarkers, imaging techniques, and therapeutic options. This, in turn, can significantly transform patient care, improve quality of life, and reduce the burden of lung diseases on healthcare systems worldwide. The promising advances documented here exemplify how dedication to scientific inquiry and technological development can propel the field forward, opening new horizons for future breakthroughs and more effective management of pulmonary pathologies.
